# Practice sensitive quality indicators in RAI-MDS 2.0 nursing home data

**DOI:** 10.1186/1756-0500-6-460

**Published:** 2013-11-13

**Authors:** Carole A Estabrooks, Jennifer A Knopp-Sihota, Peter G Norton

**Affiliations:** 1 Faculty of Nursing, University of Alberta, Edmonton, Alberta T6G 1C9, Canada; 2 Faculty of Health Disciplines, Athabasca University, Edmonton, Alberta T5K 2J8, Canada; 3 Department of Family Medicine, University of Calgary, Calgary, Alberta T2N 4N1, Canada

**Keywords:** Nursing home, Performance measurement, RAI-MDS, Quality indicators

## Abstract

**Background:**

In recent years, improving the quality of care for nursing home residents has generated a considerable amount of attention. In response, quality indicators (QIs), based on available evidence and expert consensus, have been identified within the Resident Assessment Instrument – Minimum Data Set 2.0 (RAI-MDS 2.0), and validated as proxy measures for quality of nursing home care. We sought to identify practice sensitive QIs; that is, those QIs believed to be the most sensitive to clinical practice.

**Method:**

We enlisted two experts to review a list of 35 validated QIs and to select those that they believed to be the most sensitive to practice. We then asked separate groups of practicing physicians, nurses, and policy makers to (1) rank the items on the list for overall “practice sensitivity” and then, (2) to identify the domain to which the QI was most sensitive (nursing care, physician care, or policy maker).

**Results:**

After combining results of all three groups, pressure ulcers were identified as the most practice sensitive QI followed by worsening pain, physical restraint use, the use of antipsychotic medications without a diagnosis of psychosis, and indwelling catheters. When stratified by informant group, although the top five QIs stayed the same, the ranking of the 13 QIs differed by group.

**Conclusions:**

In addition to identifying a reduced and manageable set of QIs for regular reporting, we believe that focusing on these 13 practice sensitive QIs provides both the greatest potential for improving resident function and slowing the trajectory of decline that most residents experience.

## Background

Increasing numbers of older adults, primarily due to advanced age and frailty, are in need of nursing home (NH) care. At the same time, concerns about the quality of care provided to this vulnerable population persist. As a means of measuring and evaluating NH care, a standardized data collection and monitoring system, the Resident Assessment Instrument – Minimum Data Set 2.0 (RAI-MDS 2.0) was developed by the Centre for Medicare and Medicaid in the US. This system is now used in several countries including Canada. It allows for a valid, reliable, and standardized assessment of resident outcomes measured at the person level over time [1]. The use of standardized data, such as the RAI-MDS 2.0, makes it possible to define, compare, monitor, and report quality indicators (QIs) for clinical planning and decision making in NHs [2]. Although the RAI-MDS 3.0 is now used in the US, at present all jurisdictions in Canada use 2.0 with no immediate plans to change.

In our ongoing program of research, Translating Research in Elder Care (TREC), we focus on improving the quality and safety of care delivered to residents of NHs. Protocols for this program have been published elsewhere [3,4]. Briefly, TREC closely follows a representative cohort of urban nursing homes in the Canadian Prairie Provinces, capturing RAI-MDS 2.0 data from those nursing homes from 2007 onward. As part of this research, the Safer Care for Older Persons [in residential] Environments (SCOPE) was developed with the goal of engaging frontline staff to become involved in the quality improvement process [5].

### What is a quality indicator?

A QI is a computed measure based on a clinical outcome that is believed to be reflective of the quality of care. In other words, QIs are used as proxy or surrogate measures for quality of care. Outcomes can be undesirable, such as falls or pressure ulcers, or they may be desirable such as physical independence or improved continence. QIs were central in the original conceptualization of the RAI-MDS 2.0 assessment system. Public reporting of QIs has been done for many years in the US and is beginning to be used in Ontario long-term care [6]. Public reporting is thought to be a driver of improved quality either through consumer empowerment, or by 'naming and shaming’ [7]. But more importantly, QIs give individual facilities or operators a standardized and comparable measure by which to target and monitor quality improvement activities. When reported with transparency, poor performers can identify facilities with good performance, and seek to learn from them. Researchers can use QIs as a metric to shed light on the effects of ownership, funding, policy, care culture, and other factors.

Some QIs are strictly cross-sectional (*e.g.,* use of indwelling catheters), while others use consecutive assessments to identify individual-level improvement or decline. Central to QI construction is the issue of risk adjustment, which arises from understood risk factors associated with poor outcomes, and these risk factors being unevenly distributed among facilities. Risk-adjusted QIs are designed to allow comparison of facility results with those of other facilities and to overall populations of interest. They take into account differences in the risk profiles of resident populations within individual facilities [2]. Methods for developing RAI-MDS 2.0 based QIs for use in NHs have been developed in the US [8] and have been applied in Canadian settings [9]. More recently 3^rd^ generation risk adjustment techniques have been adopted [10,11].

### Practice sensitive QIs

In Canada, there are 35 validated QIs identified in the RAI-MDS 2.0 system; however, not all of them are equally sensitive to changes in practice, be it nursing, medical, allied or combined interventions. As our intent in the TREC program of research is to work with *modifiable* outcomes, we aimed to develop a set of what we term *practice sensitive QIs*. Similar to the SCOPE project [12], we intend to use the list of practice sensitive QIs and assess them for strength of evidence that would support developing or refining interventions in the NH population. This paper describes the process used to identify and develop this list of practice sensitive QIs.

## Method

We began with the list of the 35 Canadian 3^rd^ generation quality indicators for RAI-MDS 2.0 [10]. First we sought the opinions of two experts (Poss [13,14] and Hirdes [15,16]) familiar with the selection and construction of these indicators and they identified 10 as sensitive to nursing practice, two to physician practice, and one policy/legislation intervention (see Table 1 for the RAI-MDS 2.0 codes). Second using a modified Delphi technique [17], we then recruited informants based on their reputation as experts within the NH sector. The informant groups included practicing physicians (n = 4), nurses (n = 8), and decision/policy-makers (n = 4) all of whom were familiar with the RAI-MDS 2.0. More specifically, the physician group included two geriatric specialists and two family physicians with a specific interest in geriatric medicine, the nursing group included six nationally recognized nurse scholars with active research portfolios in the NH area and two practicing geriatric clinical nurse specialists, and the decision/policy makers were either NH Directors of Care or government level policy makers with a NH portfolio. We then submitted the 13-item list to informants (n = 16) via electronic mail asking them to anonymously and independently rank the items for (1) overall “practice sensitivity” and then, (2) to identify the domain to which the QI was most sensitive (nursing care, physician care, or policy maker).

**Table 1:** RAI-MDS codes and definitions of practice sensitive quality indicators

**Quality indicator**	**RAI-MDS 2.0 assessment items**	**Definition**
**Antipsychotic use without psychosis**	O4a – Number of days during last 7 days receiving antipsychotic medication	Percent of residents on antipsychotics without a diagnosis of psychosis
**Decline in mood**	E1a – Negative statements	Percent of residents who decline in mood from symptoms of depression
	E1d – Persistent anger with self or others	
	E1f – Expressions of unrealistic fears	
	E1h – Repetitive health complaints	
	E1i – Repetitive anxious non-health complaints or concerns	
	E1l – Sad pained or worried facial expressions	
	E1m – Crying, tearfulness	
**Declining behavioral symptoms**	E4a – Wandering	Percent of residents who have declining behaviour symptoms. Where 1 or more of the indicators are greater at the target assessment than the prior assessment
	E4b – Verbally abusive	
	E4c – Physically abusive	
	E4d – Socially inappropriate behaviour	
**Fallen last 30 days**	J4a – Resident has fallen in the last 30 days	Percent of residents who have fallen in the last 30 days
**Feeding tube**	K5b – Feeding tube	Percent of residents with a feeding tube
**Indwelling catheter**	H3d – Indwelling catheter	Percent of residents with indwelling catheter
**Late loss ADL decline**	G1a – Bed mobility	Percent of residents with loss in 1 or more of the ADL late loss self-performance categories
	G1b – Transfer	
	G1h – Eating	
	G1i – Toilet use	
**Physical restraint use**	P4c – Trunk restraint	Percent of residents in physical restraints on a daily basis
	P4d – Limb restraint	
	P4e – Chair prevents rising	
**Pressure ulcer**	M2a – Stage of pressure ulcer (0 for none)	Percent of residents who have a stage 2 to 4 pressure ulcer
**Symptoms of delirium**	B5a – Easily distracted	Percent of residents with symptoms of delirium
	B5b – Periods of altered perception or awareness of surroundings	
	B5c – Episodes of disorganized speech	
	B5d – Periods of restlessness	
	B5e – Periods of lethargy	
	B5f – Mental function varies over the course of the day	
**Unexplained weight loss**	K3a – Weight loss	Percent of residents who have unexplained weight loss
**Urinary tract infections**	I2j – Urinary tract infection	Percent of residents with urinary tract infections
**Quality indicator**	**RAI-MDS 2.0 assessment items**	**Definition**
**Worsening pain**	J2a – Frequency of pain	Residents with greater pain at target assessment relative to prior assessment
	J2b – Intensity of pain	

ADL, activities of daily living.

### Ethics

Ethics and operational approvals were obtained from Health Research Ethics Board of the University of Alberta and from the participating sites respectively.

## Results

Results of the exercise are presented in Table 2. Overall, informants (n = 16) identified pressure ulcers as the most practice sensitive QI, followed by worsening pain, physical restraint use, the use of antipsychotic medications without a diagnosis of psychosis, and indwelling catheters. Additionally, the groups identified pressure ulcers, worsening pain, physical restraint use, declining behavioral symptoms, urinary tract infections, a decline in late loss activities of daily living (ADL) function (e.g., bed mobility, eating, toilet use), falls in the last 30 days, a decline in mood, and unexplained weight loss as *most* sensitive to nursing care. The use of antipsychotics – without a diagnosis of psychosis, indwelling catheters, delirium, and feeding tubes were deemed *most* sensitive to physician care. Lastly, none of the 13 QIs were deemed to be *most* sensitive to policy/decision makers. Use of antipsychotics, without a diagnosis of psychosis, followed closely by physical restraint use, and feeding tubes were the QIs identified as being the most sensitive to all domains of care (nursing, physician, and policy makers). Decline in mood and unexplained weight loss were acknowledged as the QIs least sensitive by any of the examined groups.

**Table 2:** **Results of the modified Delphi exercise to identify the *most *** practice sensitive quality indicators^*^

**Quality indicator**	**Rank order of practice sensitive QIs**^**†**^	**Number of times quality indicator chosen as *most *** practice sensitive to nursing care, physician care, or policy^*^			
		**Nursing care n (%)**	**Physician care n (%)**	**Policy maker n (%)**	**Total**^**‡**^
**Pressure ulcer**	1	**13 (81)**	1 (1)	2 (13)	16
**Worsening pain**	2	**11 (69)**	4 (25)	1 (1)	16
**Physical restraint use**	3.5	**10 (63)**^±^	3 (19)	7 (44)	20
**Antipsychotic use without psychosis**	3.5	5 (31)	**13 (81)**	3 (19)	21
**Indwelling catheter**	5	4 (25)	**10 (63)**	1 (1)	15
**Delirium**	6	4 (25)	**9 (56)**	0	13
**Declining behavioral symptoms**	7	**11 (69)**	1 (1)	0	12
**Urinary tract infections**	8.5	**10 (63)**	2 (13)	2 (13)	14
**Late loss ADL decline**	8.5	**12 (75)**	0	0	12
**Fallen last 30 days**	10	**8 (50)**	2 (13)	2 (13)	12
**Feeding tube**	11	2 (13)	**12 (75)**	4 (25)	18
**Decline in mood**	12.5	**6 (38)**	2 (13)	1(1)	9
**Unexplained weight loss**	12.5	**8 (50)**	2 (13)	0	10

ADL, activities of daily living; QI, quality indicator.

^*^Rater sample size = 16 (Physicians = 4; Nurses = 8, Decision/Policy makers = 4).

^**†**^Where ties occurred during ranking process, the rank values are represented in decimal place form.

^**‡**^Total counts of number of times QIs are seen as *most* practice sensitive can exceed the total sample size due to double rating.

^±^For example, physical restraint use was ranked most sensitive to nursing care by 10/16 raters, to physician care by 3/16 and policy makers by 7/16.

The bold numbers identify the indicators that are either sensitive to Nursing Care, Physician care, or Policy makers.

When stratified by informant group, although the top five QIs stayed the same, the ranking of the 13 QIs differed by group (Table 3). The group of nurses ranked worsening pain and antipsychotic medications without a diagnosis of psychosis as the *most* practice sensitive QIs, physicians ranked pressure ulcers *most* sensitive, while policy makers saw indwelling catheters as the *most* practice sensitive of the QIs.

**Table 3:** **Ranking and mean scores of *most *** practice sensitive quality indicators as identified by informant group^*^

**Quality indicator**	**Rank of quality indicator (mean score)**^**†**^			
	**Total (n = 16)**	**Nurse informants (n = 8)**	**Physician informants (n = 4)**	**Policy maker informants (n = 4)**
**Pressure ulcer**	1 (4.53)	3 (4.43)	1 (4.75)	2.5 (4.50)
**Worsening pain**	2 (4.47)	1.5 (4.71)	3 (4.25	4 (4.25)
**Physical restraint use**	3.5 (4.33)	5.5 (4.14)	2 (4.50)	2.5 (4.50)
**Antipsychotic use without psychosis**	3.5 (4.33)	1.5 (4.71)	4.5 (4.00)	5 (4.00)
**Indwelling catheter**	5 (4.13)	9 (3.86)	4.5 (4.00)	1 (4.75)
**Delirium**	6 (3.86)	8 (4.00)	6 (3.67)	6.5 (3.75)
**Declining behavioral symptoms**	7 (3.60)	5.5 (4.14)	7 (3.50)	12 (2.75)
**Urinary tract infections**	8.5 (3.47)	4 (4.29)	13 (2.25)	8.5 (3.25)
**Late loss ADL decline**	8.5 (3.47)	5.5 (4.14)	12 (2.50)	8.5 (3.25)
**Fallen last 30 days**	10( 3.20)	10.5 (3.29)	8.5 (3.00)	8.5 (3.25)
**Feeding tube**	11 (3.14)	13 (2.83)	8.5 (3.00)	6.5 (3.75)
**Decline in mood**	12.5 (3.00)	10.5(3.29)	11 (2.75)	13 (2.75)
**Unexplained weight loss**	12.5 (3.00)	12(3.00)	8.5 (3.00)	11 (3.00)

ADL, activities of daily living.

^*^To identify which of the quality indicators were considered *most* sensitive to practice, informants were provided the statement “This quality indicator is practice sensitive”. Responses ranged from 1 to 5 (1 = strongly disagree, 5 = strongly agree).

^**†**^Where ties occurred, the rank values are represented in decimal place form.

## Discussion

Improving the quality of care for NH residents has generated a considerable amount of attention in recent years. In response, QIs, based on available evidence and expert consensus, have been constructed and validated as reflections of both the process and outcome of care. In this paper, we described the process used for selecting and ranking the 13 practice sensitive QIs from an initial list of 35 indicators, all of which have been previously validated for use within the RAI-MDS 2.0. In addition, the QIs we have identified are congruent with those identified by the US Centre for Medicare and Medicaid Services [18] and the Health Quality Ontario [19]. These agencies utilize QIs primarily for public reporting purposes; therefore, the QIs identified have been judged to be both important and sufficiently valid (*e.g.* QIs included as parts of public reporting reflect the highest level of measurement quality). Table 4 provides a summary of the key indications of validity for the 13 QIs. While this work is based on RAI-MDS 2.0 data, we believe the process used to identify and rank the practice sensitive QIs as well as the actual indicators that we have identified will also be of interest to those using RAI- MDS 3.0.

**Table 4:** Evidence of validity for practice sensitive quality indicators

**Quality indicator**	**Evidence of quality indicator validity**
**Antipsychotic use without psychosis**	CMS: percentage of long-stay residents who received an antipsychotic medication.
**Decline in mood**	CMS: percentage of residents who have depressive symptoms.
	A (non-supportive) paper [20], reported that they found notable under-reporting; although, they agreed this QI was useful for reporting because of the clinical importance of the domain.
**Declining behavioral symptoms**	There is little yet reported to support the validity of this indicator, however it is clinically importance, and associated with resident safety.
**Delirium**	There is little yet reported to support the validity of this indicator, however it is clinically importance, and associated with resident safety.
**Fallen last 30 days**	HQO: percentage of residents who had a recent fall.
	Some data [21] suggests RAI-MDS data on falls over longer intervals (*e.g.* falls in last 180 days) may be more accurate and also cautions that falls tend to be underreported in the MDS data compared to in the chart [22].
**Feeding tube**	There is little yet reported to support the validity of this indicator, however it is clinically importance, and associated with resident safety.
**Indwelling catheter**	CMS: residents who have/had a catheter inserted and left in their bladder.
	Found to have the highest level of validity and highly recommended for use by CMS and nursing homes [22].
**Late loss ADL decline**	CMS: percentage of long-stay residents whose need for help with daily activities has increased.
	HQO: percentage of residents with increasing difficulty carrying out normal everyday tasks.
**Physical restraint use**	HQO: percentage of residents who were physically restrained.
	CMS: percent of residents who were physically restrained.
**Pressure ulcer**	HQO: percentage of residents who had worsening pressure ulcer status.
	CMS: pressure ulcer prevalence.
**Unexplained weight loss**	CMS: percentage of long-stay residents who lose too much weight. One study [23] concluded that the RAI-MDS weight loss QI is able to discriminate differences in prevalence of weight loss between facilities, suggesting concurrent validity of the QI.
**Urinary tract infections**	CMS: percentage of long-stay residents with a urinary tract infection.
	Found to have the highest level of validity and highly recommended for use by CMS and nursing homes [22].
	One study [24] comparing the RAI-MDS data for urinary tract infection (UTI), with data arising from active prospective surveillance in LTC facilities (n = 16) concluded that the RAI-MDS overestimated the number of cases. However, suggestions to use more explicit definition to reduce false positives have been instituted in 2008.
**Worsening pain**	HQO: percentage of residents with pain that recently got worse.
	The RAI-MDS *pain* QI^*^ has been found to accurately differentiate the prevalence of pain between facilities however it has been suggested that high pain prevalence scores were associated with more frequent pain assessment and appropriate pain-related care practices, as opposed to poor care quality [25].

ADL, activities of daily living; CMS, US Centre for Medicare and Medicaid Services; HQO, Health Quality Ontario; QI, quality indicator.

^*^The new CMS QI for pain is based on the self-report item of the newer MDS 3.0, and not the MDS 2.0.

## Conclusion

While we have the ability to generate all 35 indicators we believe that focusing on these 13 practice sensitive QIs, not only provides a reduced and more manageable list of QIs for reporting purposes but also have the greatest potential for functional improvement and the slowing of the trajectory of decline that most NH residents experience. Using this information, combined with data related to the frequency of “events” and our ability to measure them sufficiently well enough to see change, we will generate a short list of 3–5 topical areas in which to focus future quality improvement interventions.

## Abbreviations

QI: Quality indicator; RAI-MDS: Resident Assessment Instrument Minimum Data Set; NH: Nursing home; TREC: Translating Research in Elder Care; SCOPE: Safer Care for Older Persons [in residential] Environments; ADL: Activities of daily living.

## Competing interests

The authors declare that they have no competing interest.

## Authors’ contributions

CAE and PGN participated in conceptualizing the TREC program and in securing the grant that provided its funding. CAE and PGN conceptualized the exercise and led the data collection process. JKS drafted the initial manuscript and all authors approved the final version.
